# BDNF and miRNAs in acupuncture-regulated neuroplasticity: Emerging mechanisms and therapeutic strategies

**DOI:** 10.1097/MD.0000000000046851

**Published:** 2026-01-09

**Authors:** Jingxin Li, Lijuan Liu, Shuang Sun, Chunyan Liu, Ping Yin

**Affiliations:** a Department of Neurology, Heilongjiang Provincial Hospital, Harbin, China; b Department of Neurology, Aviation General Hospital, Beijing, China; c Department of Senior Neurology, Heilongjiang Provincial Hospital, Harbin, China; d Department of Acupuncture and Moxibustion, Heilongjiang Provincial Academy of Chinese Medicine, Harbin, China.

**Keywords:** acupuncture, BDNF, microRNA, neural plasticity, neurodegenerative diseases

## Abstract

The trend of global population aging is closely associated with a rising incidence of neurodegenerative diseases (NDs), including stroke, Alzheimer disease, Parkinson disease, and depression. These conditions, characterized by progressive neuronal loss, currently pose a significant challenge due to the lack of curative therapies. Brain-derived neurotrophic factor (BDNF) serves as a critical regulator of synaptic plasticity, a fundamental mechanism believed to underpin essential cognitive and motor functions such as learning, memory formation, and recovery. Decreased BDNF and deficits in BDNF signaling leads to the pathogenesis of NDs. Numerous studies support the therapeutic potential of acupuncture in managing NDs. Its beneficial effects are largely attributed to the ability to elevate BDNF expression and potentiate associated neurotrophic signaling. Beyond direct BDNF modulation, acupuncture exerts regulatory effects on specific micro-RNAs (miRNAs). This includes miRNAs that directly target BDNF transcripts for posttranscriptional control, as well as others that independently influence molecules critical for maintaining synaptic plasticity. The binding of acupuncture-elevated BDNF to its high-affinity receptor, Tropomyosin-related kinase B (Trk-B), initiates the activation of key downstream signaling cascades, including phosphatidylinositol 3-kinase/protein kinase B (PI3K/AKT), mitogen-activated protein kinase/extracellular signal-related kinase (MAPK/ERK) and phospholipase C-γ (PLCγ) pathways which are involved in synaptic plasticity, survival, proliferation and differentiation of neurons. In this review, we present the effects of acupuncture on BDNF, miRNAs and the downstream signal transduction pathways of BDNF in NDs and the review may partly elucidate the biological molecular mechanisms of acupuncture in the therapy of NDs.

## 1. Introduction

A defining pathological hallmark of neurodegenerative disorders (NDs) is the progressive and selective degeneration of neuronal populations. This category encompasses a spectrum of clinically heterogeneous conditions, including stroke, Alzheimer disease (AD), and Parkinson disease (PD). Notably, in the elderly population, depression can frequently serve as a clinical indicator of underlying neurodegenerative processes. Furthermore, conditions like stroke, AD, and PD are often comorbid with depression. The incidence of NDs is high and effective treatments for NDs are lacking. As a prominent member of the neurotrophin family, brain-derived neurotrophic factor (BDNF) is widely expressed throughout key brain regions like the cortex and hippocampus. It critically modulates activity-dependent synaptic plasticity, including long-term potentiation, a process fundamental to learning and memory. Beyond this, BDNF supports diverse neurobiological functions such as neuronal growth, structural remodeling, and the formation of axonal and dendritic synapses. Consequently, BDNF is recognized as a pivotal mediator of neuroplasticity, crucial for motor learning and poststroke recovery.^[1]^ Conversely, impairments in BDNF signaling or its reduced expression are strongly implicated in the pathogenesis of conditions like Alzheimer (AD) and PD.^[2,3]^ Furthermore, BDNF modulates dopamine receptor expression, underscoring its broader involvement in the pathophysiology of psychiatric conditions such as schizophrenia and mood disorders.^[4,5]^ Therefore, BDNF may be an effective therapeutic target to treat NDs.

**Figure 1.**
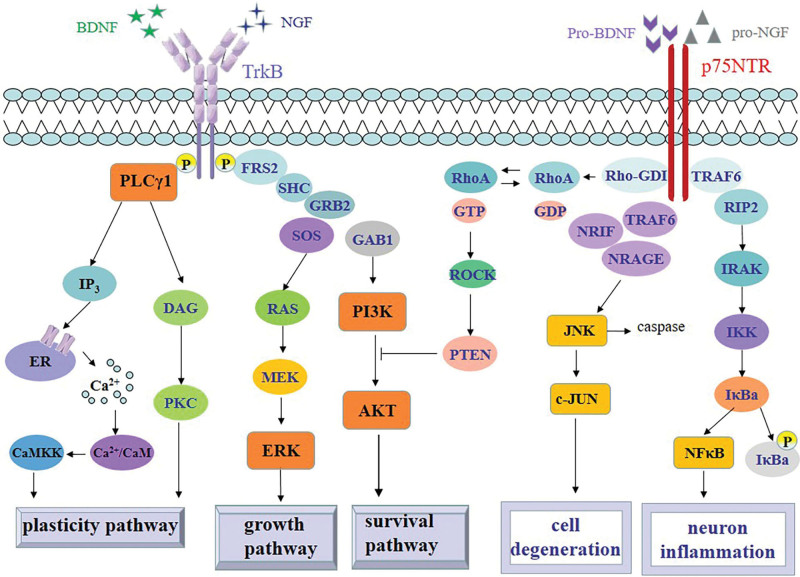
BDNF/NGF-Trk-B signaling pathways and pro-BDNF/pro-NGF-p75NTR downstream intracellular signaling cascades. BDNF binding with the extracellular domain of Trk-B leads to autophosphorylation of intracellular parts of Trk-B which provokes the activation of intracellular signaling cascades including PI3K/AKT, MAPK/ERK and PLCγ pathways. Phosphorylation of Trk-B tyrosine recruits the Shc adaptor protein, followed by recruitment of Grb2 and SOS which activates the Ras–MAPK-ERK pathway which is involved in neuron growth. Moreover, Shc–Grb2 recruits GAB1 and activates the PI3K-AKT pathway invoved in neuron survival. Moreover, phosphorylation of the remaining Trk-B tyrosine results in the recruitment of PLCγ which leads to the formation of IP3 as well as the regulation of intracellular Ca2 + and DAG. DAG can activate CaMK and PKC. PLCγ pathway has major involvements with neuronal plasticity. On the contrast, pro-BDNF or pro-NGF binding to p75NTR can cause activation of the RhoA, JNK and NFκB signaling pathways leading to dendritic spine loss, caspase release, cell death and neuroinflammation. BDNF = brain-derived neurotrophic factor, CaMK = Ca2+/calmodulin-dependent protein kinase, DAG = diacylglycerol, GAB1 = Grb2-associated binder-1, Grb2 = growth factor receptor-bound protein 2, MAPK/ERK = mitogen-activated protein kinase/extracellular signal-related kinase, NGF = nerve growth factor, PI3K/AKT = phosphatidylinositol 3-kinase/protein kinase B, p75NTR = pan-neurotrophin receptor p75, PKC = protein kinase C, PLCγ = phospholipase C-γ, Shc = Src homology 2 domain-containing, SOS = son of sevenless, TrK-B = tropomyosin-related kinase B.

Traditional Chinese acupuncture has a long history for over 2500 years and is becoming more popular worldwide. Numerous previous studies have shown that acupuncture including manual acupuncture (MA) and electroacupuncture (EA) alleviate symptoms of stroke, AD, PD and depression by increasing the levels of BDNF^[6]^ and promoting neurotrophic effects.^[7,8]^ Micro-RNAs (miRNAs) function as posttranscriptional regulators by binding to the 3’-UTR of target mRNAs, leading to their degradation or translational repression.^[9]^ Acupuncture can regulate some miRNAs which directly bind with the 3′-UTR of BDNF to regulate BDNF. Acupuncture can also affect some miRNAs which are involved in neural synaptic plasticity. Above all, acupuncture can regulate both BDNF and miRNAs to affect neuroplasticity to improve the prognosis of NDs. The mechanistic cascade involves acupuncture-elevated BDNF binding to its high-affinity Tropomyosin-related kinase B (Trk-B) receptor, which in turn activates crucial downstream signaling pathways such as phosphatidylinositol 3-kinase/protein kinase B (PI3K/AKT), mitogen-activated protein kinase (MAPK)/extracellular signal-related kinase (MAPK/ERK) and phospholipase C-γ(PLCγ) pathways. These pathways, central to neuronal health and function, are detailed in the Figure 1.^[10,11]^

We review the effects of acupuncture on BDNF, miRNAs and the downstream signal transduction pathways of BDNF in NDs. The aim of this review is to explore the biological molecular mechanisms of acupuncture in the therapy of NDs.

## 2. BDNF and miRNAs in NDs

### 2.1. The biological role of BDNF in NDs

BDNF is initiated from a precursor molecule, pre–pro-BDNF, which undergoes proteolytic cleavage to form pro-BDNF. This intermediate is further processed to yield mature BDNF.^[12]^ The biological actions of these forms diverge significantly: pro-BDNF exhibits a high affinity for the p75 neurotrophin receptor (p75NTR), triggering pathways often associated with neuronal apoptosis.^[13]^ In contrast, mature BDNF binds selectively to the Trk-B receptor on neural cells, initiating the activation of 3 principal downstream signaling cascades – the PI3K/AKT, MAPK/ERK, and PLCγ pathways. These cascades are instrumental in regulating synaptic plasticity, neuronal survival, proliferation, and differentiation.^[10,11]^

Existing research demonstrates that cerebral ischemia-reperfusion injury (CIRI) significantly reduces the population of BDNF-immunopositive neurons across critical neurogenic niches, including peri-infarct cortical regions, the striatal complex, subventricular zone (SVZ), and hippocampal formation in rat models of ischemic stroke.^[14,15]^ In rodent models of middle cerebral artery occlusion (MCAO), the up-regulation of BDNF within the nervous system promotes neuroplasticity, thereby facilitating the recovery of motor learning capabilities during poststroke rehabilitation.^[16]^ BDNF serves as a critical regulator of learning and memory processes. Notably, its levels are markedly diminished in the brains of AD patients, resulting in compromised synaptic plasticity and neuronal degeneration.^[17]^ Preclinical studies consistently report reductions in both BDNF mRNA and protein expression in AD animal models,^[18]^ with these deficits in neurotrophic signaling being closely linked to disease initiation. Treating AD with BDNF can improve AD cognitive disorder.^[19]^ Postmortem analyses of PD patients reveal decreased BDNF mRNA^[20]^ and protein^[21]^ expression in the substantia nigra (SN), implicating dysfunction of the BDNF/Trk-B signaling axis in the pathogenesis of PD. Notably, clinical interventions involving antiparkinsonian medications have been shown to elevate BDNF concentrations.^[22]^ Additionally, exercise-based therapies induce corticomotor excitation and augment BDNF levels, thereby enhancing neural plasticity in individuals with PD.^[23]^ Reductions in BDNF levels and Trk-B phosphorylation are observed in the prefrontal cortex and hippocampus of both depressed humans and animal models.^[24–27]^ Individuals carrying the BDNF Val66Met polymorphism exhibit heightened susceptibility to stress and depression, attributable to impaired BDNF secretion.^[28]^ Furthermore, the BDNF/Trk-B system plays a modulatory role in hippocampal neurogenesis, exerting differential effects within the dentate gyrus and SVZ.^[29]^ Increasing evidences demonstrate that BDNF-mediated stimulation of hippocampal neurogenesis is a key mechanism underlying the alleviation of depressive symptoms.^[30–32]^

In summary, downregulation of BDNF and its downstream pathways are important mechanisms in NDs pathogenesis, and increasing BDNF expression can be an effective treatment attributing to improved synaptic plasticity and survival of neurons.

### 2.2. miRNAs regulate neuron plasticity in NDs

Emerging research underscores the pivotal role of miRNAs in mediating neuroprotective responses and modulating synaptic plasticity processes within neurodegenerative contexts. Specifically, miRNAs orchestrate synaptic transmission dynamics and plasticity mechanisms in both the hippocampal formation and neocortical areas, consequently exerting influence on memory encoding and consolidation pathways.^[33]^ miR-133b can modulate the expression of specific genes, facilitates structural remodeling of neurites, and enhances neurological recovery in rats following MCAO.^[34]^ Intracerebroventricular administration of miR-494 agomir confers substantial neuroprotective and regenerative benefits in MCAO models. This therapeutic approach markedly attenuates neuronal apoptotic processes and reduces cerebral infarct dimensions during the acute ischemic phase, while simultaneously enhancing axonal reorganization and facilitating long-term sensorimotor functional restoration across the rehabilitation continuum.^[35]^ In animal models of AD, elevated expression of miR-338-5p and miR-181 confers protection against deficits in synaptic plasticity, learning capacity, and memory consolidation.^[36,37]^ miR-132 plays a multifaceted role in neuronal development, participating in axonal elongation, cell migration, and plasticity regulation; notably, it exhibits the most pronounced reduction among all miRNAs in the brains of AD patients. Genetic ablation of miR-132 in murine models accelerates amyloid-beta deposition, compromises memory function, and exacerbates tau-related pathology.^[38]^ Therapeutic modulation of miR-132 expression effectively ameliorates clinical manifestations, reduces pathological severity, and represents a promising avenue for disease intervention.^[39]^ Besides, miR-132 dysregulation is also associated with frontolimbic network function and structure which is a critical circuit governing emotional regulation and memory processes in major depressive disorder.^[40]^ Genetic ablation of miR-137 in murine models of depressive disorders induces significant deficits in synaptic transmission and plasticity mechanisms, concurrently manifesting anxiety-like and depression-like behavioral phenotypes.^[41]^ Additionally, late-life depressive symptoms are linked to reduced miR-484 levels in the prefrontal cortex, where this miRNA modulates synaptic transmission pathways.^[42]^ Moreover, miR-99a potentially regulates synaptic plasticity mechanisms within the hypothalamic region and emerges as a promising therapeutic target for alleviating depressive symptoms associated with peri- and postmenopausal states.^[43]^ Some miRNAs also play a negative role in neuroprotection and synaptic plasticity in NDs. Specifically, miR-124 and miR-181a are directly linked to deficits in synaptic integrity.^[44,45]^ miR-124, which exhibits high neuron-specificity, plays a dual role by modulating apoptotic processes while concurrently impairing synaptic plasticity and axonal regeneration in cerebral ischemic injury.^[46]^ Similarly, miR-181a influences synaptic function during stroke recovery; overexpression of miR-181a results in dendrites with reduced spine density and diminished spine size.^[47]^ In hippocampal CA1 dendrites of stroke model rats, miR-134 is abundantly expressed and negatively modulates LIM domain kinase 1 (LIMK1), thereby exacerbating damage to synaptic-dendritic plasticitye.^[48]^ Furthermore, alleviation of depression-like behaviors involves regulatory mechanisms mediated by the synaptic miRNA miR-134 in the basolateral amygdalar complex.^[49]^ Separately, elevated expression of miR-142-5p and miR-134-5p triggers synaptic dysfunction associated with Aβ-mediated pathophysiology contributing to AD pathogenesis.^[50,51]^ Elevated expression of miR-153 offers novel perspectives on the molecular basis of presynaptic plasticity deficits, indicating that chronic cerebral hypoperfusion compromises synaptic vesicle fusion events via miR-153-mediated downregulation of key vesicle-associated protein complexes.^[52]^ Similarly, the synergistic action of miR-92a and miR-485, in line with histone-modifying enzymes, potentially alters gene regulatory networks, thereby modulating the neuroadaptive plasticity of the dorsal hippocampus in response to stressful conditions.^[53]^

In summary, miRNAs are involved in neural plasticity and neuroprotection. Regulating miRNAs expression can affect the pathogenesis, alleviates severity, and finally affects a cure of NDs.

### 2.3. miRNAs regulate BDNF to influence NDs

The miRNA- modulated BDNF signaling cascades are critically implicated in the pathogenic mechanisms underlying NDs. Evidence reveals that the 3′-UTR of BDNF serves as a prominent target for miRNAs, with in silico predictions indicating the presence of up to 17 binding sites that could be recognized by approximately 26 distinct miRNA species.^[54]^

Dual-luciferase reporter assays have identified miR-210 as a critically involved miRNA in ischemic stroke pathology, demonstrating its capacity to regulate BDNF expression and highlighting its potential as a therapeutic target for stroke intervention.^[55]^ Furthermore, the single nucleotide polymorphism rs7124442 located within the 3′-UTR of BDNF may confer protective effects in ischemic stroke patients through modulating miR-922-mediated regulatory control over BDNF expression.^[56]^ Similarly, bioinformatic analyses of MCAO brain tissues reveal that miR-10b-5p directly interacts with specific 3′-UTR binding sites on BDNF transcripts, thereby exerting a negative regulatory effect on BDNF expression levels.^[57]^ Inhibition of miR-155 can increase BDNF to protect against ischemic brain injury^[58]^ and antidepressant functions.^[59]^ Besides miR-155, another study reports that miR-1, miR-10b, and miR-191 directly suppress BDNF expression through targeting high-affinity binding sites within the BDNF 3′-UTR region.^[60]^

Elevated expression of miR-206 is consistently observed in both AD patients and experimental models, where this microRNA exacerbates memory deficits through transcriptional repression of BDNF.^[61,62]^ It has been reported that miR-30a-5p suppresses BDNF expression in the prefrontal cortex^[54]^ and may be a potential biomarker for PD.^[63]^ In atrazine-induced PD models, miR-7 expression is significantly elevated in the rat brain, where it modulates BDNF levels via an autoregulatory feedback mechanism.^[64]^ miR-206 has been established as a key regulatory factor involved in depression pathogenesis, directly targeting BDNF in both in vivo and in vitro experimental settings.^[65]^ Therapeutic inhibition of miR-124 represents a potential strategy for depression treatment, as it activates the BDNF–Trk-B signaling cascade within the hippocampal region.^[66]^ Furthermore, miR-16, which binds to the 3′-UTR region of BDNF transcripts,^[67]^ facilitates the antidepressant effects of fluoxetine by orchestrating hippocampal neurogenesis processes.^[68]^

Therefore, according to the results of the existing experimental studies, interfering with miRNA to regulate BDNF expression can be a strategy for NDs treatment.

## 3. The downstream pathways of BDNF in NDs

BDNF binds with Trk-B receptor and then stimulates 3 downstream signaling pathways PI3K/Akt, MAPK/ERK and PLCγ which are involved in neuronal synaptic plasticity, survival, proliferation and differentiation and are very important for NDs treatments (Fig. 1).

### 3.1. PI3K/AKT signaling pathway

The binding of BDNF to its high-affinity Trk-B receptor initiates the activation of the PI3K/AKT signaling cascade. This pathway orchestrates multiple critical cellular processes, including neuronal survival, autophagy regulation, neurogenesis, cellular proliferation and differentiation, as well as synaptic plasticity modulation. The PI3K/AKT pathway mediates a wide spectrum of biological functions through phosphorylation events and protein complex formation with various downstream effectors. Key molecular targets include mammalian target of rapamycin (mTOR), forkhead box O (FoxO) transcription factors, glycogen synthase kinase-3β (GSK-3β), and nuclear factor erythroid 2-related factor 2 (Nrf2), among others.^[69,70]^ As a primary downstream effector of PI3K/AKT signaling, mTOR plays a pivotal role in neuronal dendritic development, dendritic spine formation, and the regulation of autophagic processes. PI3K/AKT modulates apoptosis through inhibiting B-cell lymphoma-2 (BCL-2)-antagonist of cell death (Bad), Bcl-2-like protein 11 (BIM), caspase-9 and FoxO1. PI3K/AKT inhibits oxidative stress through modulating Nrf2, thereby facilitating transcriptional activation of genes encoding detoxification enzymes and antioxidant proteins. IKK/IκBα/NF-κB pathway, the other downstream pathway of AKT, is a vital pathways for cell survival.^[71]^ Baicalin promotes astrocyte to release BDNF and modulates both the BDNF-Trk-B/PI3K/AKT and MAPK/ERK1/2 signaling pathways to attenuate injury induced by oxygen–glucose deprivation(OGD) in a neuron-astrocyte co-culture model.^[72]^ Herba Total Alkali LHA exerts neuroprotective effects in CIRI models by elevating BDNF expression and subsequently regulating the PI3K/AKT signaling cascade.^[73]^ The PI3K/AKT/mTOR signaling axis undergoes significant alterations in AD and PD, leading to autophagy disruption and Aβ accumulation. Pharmacological inhibition of mTOR activity enhances autophagic flux, demonstrating protective effects against neuronal cell damage.^[74]^ AKT phosphorylates GSK-3α at Ser21 and GSK-3β at Ser9, leading to suppression of GSK-3-kinase activity, and therefore weakens tau protein hyperphosphorylation and neurofibrillarytangles (NFT) formation.^[75]^ Catalpol exerts antidepressant efficacy in chronic unpredictable mild stress (CUMS) models through up-regulation of the PI3K/AKT/Nrf2/HO-1 signaling axis, thereby promoting neuroprotective effects, enhancing neurotrophic support, and augmenting antioxidant mechanisms within the hippocampal region.^[76]^

### 3.2. MAPK/ERK signaling pathway

ERK1/2 represent key constituent members of the MAPK family. The activation of the MAPK signaling cascade entails a sequential phosphorylation relay involving 4 core components: small GTPases (including Ras and Rac), MAPK kinase kinases (such as Raf or MEKK), MAPK kinases (MEK), and finally the MAPKs themselves. This kinase cascade orchestrates fundamental cellular processes including proliferation, differentiation, and survival in proliferative cells^[77]^ and neural induction, neural patterning, neurogenesis, neurite outgrowth in neurodevelopment.^[78]^ In postmitotic neurons of the adult mammalian brain, MAPKs remain expressed and functionally active, where they dynamically respond to fluctuating synaptic inputs. These kinases modulate neuronal excitability and synaptic plasticity through both transcription-dependent mechanisms and direct posttranslational modifications.^[79]^

Cholic acid confers cytoprotective effects on neurovascular units against OGD-induced injury by augmenting the secretion of BDNF and simultaneously activating both the BDNF-Trk-B-PI3K/AKT and BDNF-Trk-B-MAPK/ERK signaling cascades.^[80]^ Moreover, empirical studies demonstrate that disturbances of MAPK/ERK pathways contributes to the pathogenesis of Aβ and tau protein-related neurodegenerative disorders, including AD, Pick disease, progressive supranuclear palsy, and corticobasal degenerative diseases.^[81–83]^ The ERK signaling pathway activity is markedly suppressed in the prefrontal cortex and hippocampus, which represent key neural substrates involved in depression pathogenesis. Experimental inhibition of the ERK cascade within these specific brain regions elicits behavioral manifestations resembling depression. Furthermore, multiple classes of antidepressant medications alleviate depressive symptomatology, at least partially, through restoration of the impaired ERK signaling function.^[84]^ Chronic peripheral administration of BDNF enhances ERK and cAMP-related element-binding protein(CREB) phosphorylation which is a major transcription factor mediating neuronal plasticity in depressed mouse hippocampus and produces antidepressant effects.^[85]^ Focal administration of BDNF through intracranial microinjection into either the midbrain or hippocampal regions replicates the antidepressant efficacy observed with systemic BDNF delivery,^[86,87]^ while concurrently enhancing site-specific phosphorylation of ERK.^[87]^ Pharmacological inhibition of ERK signaling using the selective inhibitor U0126 completely abolishes the antidepressant actions elicited by intrahippocampal BDNF infusion.^[86]^ But the perception that ERK1/2 opposes cell death is challenged. In ischemic conditions, for instance, noxious stimuli-induced ERK activation can paradoxically promote either cell survival or death, with the ultimate outcome being contingent upon specific experimental parameters including ischemia duration and model organism characteristics.^[88]^ Elevated levels of phosphorylated ERK have been detected in postmortem brain extracts from AD patients^[89]^ and in SN of PD patients.^[90]^ Studies utilizing dopaminergic B65 cell lines reveal that 6-OHDA-elicited cell death necessitates sustained ERK activation, and that MEK inhibition provides significant protection against such cytotoxicity.^[91]^ Also MPTP, a extensively characterized neurotoxin employed in PD modeling, provokes ERK activation, whereas ERK suppression confers cellular rescue.^[92,93]^ The roles of ERK in neuron survival is very complicated.

### 3.3. PLCγ signaling pathway

PLCγ serves as a critical signal transduction molecule that mediates the conversion of extracellular stimuli into intracellular signaling events through the production of key second messengers, including DAG and IP3. DAG acts as a potent activator for either PKC or transient receptor potential cation channels (TRPCs), whereas IP3 triggers the release of calcium ion efflux from intracellular storage sites, thereby initiating downstream signaling cascades. Second messenger systems play a pivotal role in neuronal development by modulating key processes such as neurite extension, cellular migration, axonal guidance, and synapse formation. These structural adaptations are facilitated through precisely regulated intracellular calcium dynamics following activation of the receptor tyrosine kinase-PLCγ signaling cascade.^[94]^ Consequently, dysregulation of PLCγ-mediated signaling pathways contributes to multiple neuronal developmental abnormalities and has been implicated in the pathogenesis of various neurological conditions, including NDs, major depressive disorder, and epilepsy.

Tejeda designs a blood–brain barrier permeable peptide incorporating the full-length Trk-B receptor. This innovative therapeutic strategy inhibits the internalization of Trk-B receptors from neuronal membranes, thereby enhancing neuronal survival through PLCγ-dependent signaling mechanisms and maintaining the transcriptional activity of downstream targets such as CREB and MEF2 in stroke models.^[95]^ Theanine has a protective effect associated with its interaction with monoamine neurotransmitters and up-regulates the expressions of GluR2 mRNA and PLC-γ1 mRNA in CIRI rats.^[96]^ The protective PLCγ2-P522R variant identified in AD enhances cellular survival, amplifies acute inflammatory responses, and potentiates phagocytic activity in macrophages through TREM2-associated signaling pathways.^[97]^ Huangpu Tongqiao capsules can ameliorate learning and memory deficits in AD rat models, potentially via modulation of the EGFR-PLCγ signaling axis.^[98]^ Genetic ablation of PLCγ1 in the forebrain induces manic-like behavioral phenotypes in experimental models.^[99]^ Repeated inescapable shock selectively decreases the expression of PLCβ1 and PLCγ1 isozymes in learned helplessness rats brains, with these reductions being implicated in the pathophysiology of depression and related stress disorders.^[100]^ The dipeptide BDNF mimetic GSB-106 modulates Trk-B-mediated activation of MAPK/ERK, PI3K/AKT, and PLCγ pathways, thereby reversing depressive-like behaviors and restoring hippocampal neuroplasticity in rodent models of depression.^[101]^ Chronic administration of antidepressant agents promotes Trk-B-dependent activation of phospholipase-PLCγ1 and enhances phosphorylation of the transcription factor CREB.^[102]^

## 4. Acupuncture regulating BDNF, microRNA and BDNF downstream pathways to treat NDs

As described above, accumulating data have indicated that BDNF binding with the Trk-B receptor to activate its downstream pathway to regulate synaptic plasticity and the pathophysiology in NDs. miRNAs are implicated in neural synaptic plasticity mechanisms and directly bind to the 3′-UTR of BDNF transcripts to modulate BDNF expression, thereby facilitating neuroplastic processes. Hence, miRNA/BDNF and BDNF downstream pathways regulatory networks may emerges as a pivotal mechanism governing neural plasticity. Accumulating studies demonstrates that acupuncture intervention produces beneficial clinical outcomes in NDs. Accordingly, we summarize current research elucidating acupuncture’s modulation of BDNF expression, miRNA profiles, and downstream signaling cascades, as comprehensively summarized in Tables 1 and 2.

**Table 1:** miRNAs and target genes regulated by acupuncture in treating NDs.

Disease	Model/tissue	Acupoints	Intervention	miRNAs	Main results	Reference
Stroke	Male C57B6J/L mice, MCAO, brain cortex tissues	GV20, GV14, ST36, LI11	EA, 1 mA, 2 Hz, 20 min, QD for 7 d	mmu-miR-434-3p-Prc1, mmu-miR-453-Prc1, miR-425-5p-Cdk1 mmu-miR-1186b-Prc1	Alleviate neurological deficits in the affected limbs and reduce infarct area.	[103]
	Male SD rats, MCAO, peri-ischemic striatum, Primary stem cells treated with OGD/R	LI11, ST36	EA, 1 mA, 1/20 Hz, 30 min, QD for 21 d	miR-146b	Improve neurological deficits, promote neural stem cells differentiation into neurons.	[104]
	Male SD rats, CIRI, SVZ and hippocampus	TE5, ST36	EA, 1 mA, 20 Hz, 30 min, QD for 7 d	miR-223	Up-regulate NESTIN and NOTCH1 and down-regulate PTEN expression.	[105]
	Male SD rats, MCAO, penumbra brain tissues, Primary neurons treated with OGD	GV20	EA, 1–2 mA, 2/10 Hz, 30 min, QD for 5 d	miR-132	Enhance neurobehavioral functional recovery, promote neurite outgrowth and suppress SOX2 expression.	[106]
	Male SD rats, MCAO, penumbra brain tissues	GV20	EA, 1 dilatational wave, 30 min, QD for 5 d	miR‐191a‐5p	Increase cell viability, decrease apoptosis, reduce infarct volumes, and improve neurological scores	[107]
	Male Wistar rats, MCAO, cortex	GV26, PC6	EA, 3 mA, 2 Hz, 1 min, Bid for 1–3 wk	rno-miR-206-3p, rno-miR-3473, rno-miR-6216, rno-miR-494-3p	Increase cerebral blood flow, alleviate neurological impairment and activate the VEGF signaling pathway.	[108]
	Male SD rats, MICD, hippocampus tissue	GV20, GV24	EA, 0.2 mA, 1/20 Hz, 30 min, QD for 14 d	miR-134	Reduce injured volumes, rescue synaptic-dendritic loss and negatively regulate LIMK1 to enhance synaptic-dendritic plasticity.	[48]
	Male SD rats, MCAO, penumbra brain tissues	GV20	EA, 1–2 mA, 2/10 Hz, 30 min, QD for 5 d, followed by 2 d of rest, for a period of 4 wk.	pirb mRNA, miR-181b	Enhance axon regeneration	[109]
	Male SD rats, MCAO, peri-infarct cortex	TE5, ST36	EA, 1 mA, 20 Hz, 30 min, QD for 7 d	miR-223	Decrease neurological deficit score and infarct volume. Decrease the levels of NLRP3, caspase-1, IL-1β, and IL-18	[110]
	Male SD rats, MCAO, peri-infarct cortex	LI11, ST36	EA, 4V, 1/20 Hz, 30 min, QD for 7 d	miR-9	Decrease neurological deficit score and infarct volume. Reduce NF-κB p65, TNF-α and IL-1β.	[111]
	Male SD rats, ICH, perihemorrhagic penumbra	GV20, GB7	MA, GV20-penetrating-GB7 acupuncture for 30 min, QD for 3 d	miR-34a-5p	Accelerate microglia M2 polarization through targeting Klf4.	[112]
AD	Male SAMP10 mice, hippocampus	CV17, CV12, CV6, ST36, SP10	MA, each point except SP10 was needled for 30 s with reinforcing method. SP10 was needled for 30 s with reducing method, QD for 15 d with a rest at the 8th day.	Hsp84, Hsp86, YB-1	Retard molecular events with aging.	[113]
	APP/PS1 mice, hippocampus	GV20	EA, 1 mA, 2/15 Hz, 30 min, 5 d/wk, 2 d rest for a period of 4 wk	NDRG2	Ameliorate cognitive impairments and suppress GFAP.	[114]
PD	C57BL/6J mice transformed with SNCA*A53T gene, striatum	GB34, LR3	EA, 1 mA, Hz, 30 min, QD for 2 wk	miRNA-124	Reduce the aggregation of α-synuclein protein in striatum. Up-regulate the expression of p-CREB and BDNF protein.	[115]
Depression	Male Wistar rats, CUMS, serum	GV20, GV29	EA, 1 mA, 2 Hz, 20 min, QD for 4 wk	miR-383-5p miR-764-5p	Improve behavioral indexes. Promote neurotrophy and inhibit abnormal apoptosis of neurons.	[116]
	Male SD rats, CUMS, hippocampus and serum	GV20, GV29	EA, 1 mA, 2 Hz, 20 min, QD for 4 wk	miRNA-16	Improve base levels of sucrose preference and exploratory behavior. Decrease SERT protein and increase 5-HT in the hippocampus.	[117]
	Male SD rats, CUMS, hippocampus and middle raphe nucleus	Heyi, Badagan and Xin acupoints	MA, QD for 28 d	miRNA-16	Improve the depressive state. Up-regulate the expression of p11 and tPA proteins and BDNF mRNA.	[118]

5-HT = 5-hydroxytryptamine, AD = Alzheimer disease, BDNF = brain-derived neurotrophic factor, CIRI = cerebral ischemia-reperfusion injury, CREB = cyclic adenosine monophosphate response elements binding protein, CUMS = chronic unpredictable mild stress, EA = electroacupuncture, GFAP = glial fibrillary acidic protein, ICH = intracerebral hemorrhage, IL = interleukin, MA = manual acupuncture, MCAO = middle cerebral artery occlusion, MICD = middle cerebral artery occlusion induced cognitive deficit, miRNAs = micro-RNAs, NDRG2 = N-myc downregulated gene 2, NLRP3 = nucleotide-binding domain-like receptor family, OGD/R = oxygen–glucose deprivation and reperfusion, PD = Parkinson disease, PTEN = phosphatase and tensin homolog, QD = daily, QOD = every other day, SAMP10 = senescence-accelerated mouse, SD rat = Sprague Dawley rat, SERT = 5-HT transporter, SOX2 = the sex-determining region Y-box 2, SVZ = subventricular zone, TNF-α = tumor necrosis factor-alpha, VEGF = vascular endothelial growth factor.

**Table 2:** The downstream neuropathways of BDNF regulated by acupuncture in treating NDs.

Disease	Model	Acupoints	Intervention	Signal pathway	Main results	Reference
Stroke	Male SD rats, MCAO or MCBVAO	GV20, GV14, LI11, ST36	EA, 2/15 Hz, 30 min, QD for 14 d	Increase the expression of BDNF/Trk-B	Promote motor recovery	[119]
	Male C57BL/6 mice, MCAO and grafted Trk-B-MSCs into the ischemic penumbra	GV20, GV14	EA, 2 v, 2 Hz, 20 min, QD for 10 d	Promote the expression of BDNF and NT-4	Induces the differentiation of Trk-B-MSCs, and improve motor function.	[120]
	Male C57BL/6 and C57BL/6-Tg (CH-EGFP) mice, MCAO and transplanted with Bone marrow MSCs	GV20, GV14	EA, 2 V, 2 Hz, 20 min, QD for 12 d	Increase the expression of BDNF/CREB in the neuroblasts of the striatum.	Enhance BDNF and NT-4 expression in the SVZ, striatum and hippocampus.	[121]
	Male SD rats, MCAO	GV20	MA, 1 min, at a frequency of 200 rpm, 2 h, QD for 3 wk	Increase BDNF/Bcl-2 expression	Increase BDNF, S100b, and GFAP-positive cells	[122]
	Male SD rats, MCAO	GV20	EA, 3 mA, 2/20 Hz, 30 min, QOD for 14 d	Increase the expression of BDNF/Trk-B	Elevation of BDNF neuron proliferation	[123]
	Male SD rats, MCAO	LI11, ST36	EA, 1 mA, 1/20 Hz, 30 min	Activation of the PI3K/AKT pathway	Elevation of BDNF, GDNF, Bcl-2/Bax ratio anti-apoptosis	[124]
	SD rats, CIRI	GV20, GV24	MA, 20 min, QD for 2 wk	Activation of the PI3K/AKT pathway	Elevation of VEGF, GAP-43 and synuclein	[125]
	Male SD rats, MCAO	GV20, LI4, LR3	EA, 3 mA, 2/20 Hz, 30 min, QD for 1–2 d	Increase expression of p-AKT protein	Evation of CD34^+^ endothelial progenitor cell	[126]
	Male Wistar rats, MCAO	GV20, GV26	EA, 3 mA, 3/20 Hz, 60 min	Activation of AKT	Depression of caspase-9 anti-apoptosis	[127]
	Male SD rats, MCAO	GV26, CV24	EA, 3 mA, 4/16 Hz, 30 min	Activation of the PI3K pathway	Neuroprotection	[128]
	Male SD rats, MCAO	GV26, CV24	EA, 1/3 V, 4/16 Hz, 30 min	Activation of the TrkA-PI3K pathway	Neuroprotection	[129]
	SD rats, MCAO	GV26, CV24	EA, 1/3 V, 4/16 Hz, 30 min	Activation of the TrkA-PI3K pathway	Depression of NO, nNOS and iNOS	[130]
	Male SD rats, MCAO	LI11, ST36	EA, 1mA, 1/20 Hz, 30 min	Activation of the PI3K/AKT pathway	Elevation of BDNF, GDNF, Bcl-2/Bax ratio anti-apoptosis	[124]
	SD rats, LCCA ligation	GV20, GV14, LI11, KI1	MA and EA, EA: 3/5 V, 5/10 Hz, 10 min, QD for 1, 3, 7, and 21 d respectively	Activation of the PI3K/AKT pathway	Neuroprotection	[131]
	Male SD rats, MCAO	LI11, ST36	EA, 0.2 mA, 1/20 Hz, 30 min, QD for 3 d	Activation of the mTORC1-ULK1 complex-beclin1 pathway	Depression of microtubule associated protein 1 light chain 3 beta II/I, ULK1, autophagy related gene 13 and Beclin1 anti-autophagy	[132]
	Male SD rats, CIRI	GV20, GV24	EA, 2 mA, 1/20 Hz, 30 min, QD for 8 d	Activate the PI3K/AKT/mTOR pathway	Improve CIRI rat learning and memory impairment	[133]
	Male SD rats, CIRI	GV20, LI4, LR3	EA, 1 mA, 2/20 Hz, 30 min, QD for 3 d	Activate the PI3K/AKT and NF-κB signaling pathways through TREM2	Attenuate inflammatory injury following CIRI	[134]
	Male SD rats, MCAO	LI11, ST36	EA, 4/20 Hz, 30 min, QD for 3 d	Activation of the PI3K/AKT pathway	Elevation of PI3K, p-AKT, p-Bad and Bcl-2, depression of Bax, caspase-3-positive expression anti-apoptosis	[135]
	Male SD rats, MCAO	GV20, GV14	EA, 2.7–3.0 mA, 5 Hz, 25 min, QD for 2 d	Activation of MAPK/ERK kinase, the ERK1/2 pathway	Elevation of BDNF, pRaf-1, pp90RSK, p-Bad. Depression of caspase-3 protein	[136]
	Male SD rats, MCAO	LI11, ST36	EA, peak 6 V, 1/20 Hz, 30 min, QD for 3 d	Activation of the ERK1/2 pathway	Elevation of p21 or p27. Depression of cyclinD1, CDK4, cyclin E and CDK2. Neural cell proliferation	[137]
	Male SD rats, MCAO	GV20	EA, 1.0 mA, 2/15 Hz, 30 min	Activation of the ERK1/2 pathway	Elevation of CB1 neuroprotection	[138]
	Male SD rats, MCAO	LI11, ST36	EA, 1/20 Hz, 30 min	Activation of the ERK1/2 pathway	Elevation of Ras, cyclin D1 and CDK4. Neural cell proliferation.	[124]
	Male SD rats, MCAO	GV20, GV16	EA, 2.7–3.0 mA, 5 Hz and 25 Hz, 25 min, QD for 7 d	Activation of the p38 MAPK/CREB pathway	Decrease reactive astrocytosis	[139]
	Female SD rats, MCAO	GV20, GV24	EA, 1/20 Hz, 30 min, QD for 7 d	Inactivation of the CaM-CaMKIV-CREB pathway	Inactivation of the CaM-CaMKIV-CREB pathway	[140]
	Neonatal SD rats, CCAO	GV20, ST36	EA, 1 mA, 2 Hz, 20 min	Activation of the CREB/BDNF pathway	Oligodendrogenesis	[141]
	Male Wistar rats, homologous blood emboli injection of internal carotid artery	ST36	MA, QD for 14 d	Activation of the cAMP/PKA/CREB pathway	Activation of LTP	[142]
	Male SD rats, MCAO	GV20, HT7	MA, EA, 30 mW, 100 Hz, QD for 14 d	Enhance cholinergic system	Elevation of CREB, BDNF, and Bcl-2. Depression of Bax anti-apoptosis.	[143]
	Male SD rats, MCAO	LI11, ST36	EA, 1 mA, 4/20 Hz, 30 min, QD for 3 d	Modulation of the ERK/JNK/p38 signal pathway	Elevation of caspase-3, growth factor midkine. Depression of Bcl-2 anti-apoptosis.	[144]
	Male SD rats, MCAO	GV20, GV14, GV26	MA, 30 min/time, 7 times	Inactivation of the MAPK/ERK pathway	Elevation of Bcl-2. Depression of Bax anti-apoptosis	[145]
AD	APP/PS1 mice	GV20	EA, 1/20 Hz, 30 min, QD for 4 wk	Up-regulation of Trk-B and downregulation p75NTR	Ameliorate cognitive impairments, elevate BDNF, reduce the aberrant overexpression of Aβ1–42, and inhibit neuronal apoptosis	[146]
	Male rats administered with scopolamine	GV20	MA, 5 min, QD for 14 d	Enhance cholinergic system–CREB–BDNF pathway	Restore the expression of CHT1, VAChT, BDNF and CREB mRNA in the hippocampus	[147]
	telomerase-deficient (Terc⁻/⁻) mice	ST36	MA, 30 min, QD for 4 d	Up-regulate BDNF, Trk-B, p75NTR, AKT, and ERK1/2 in the dentate gyrus and hippocampus	Up-regulate BDNF, Trk-B, p75NTR, AKT, and ERK1/2 in the dentate gyrus and hippocampus	[148]
	APP/PS1 mice	GV20	EA, 1/20 Hz, 30 min, 5 d/wk, and 2 d rest for a period of 4 wk	Induce AMPK and AKT and inhibit mTOR	Increase glucose metabolism in specific brain regions, decrease the accumulation of Aβ	[149]
PD	Male C57BL/6N mice induced by MPTP	GV20, GV29	EA, 1.5 mA, 2 Hz, 10 min, QD for 7 wk	Restore the Trk-B/AKT/ERK1/2 neurotrophic signaling pathway	Restore the dopaminergic neuronal function and up-regulate BDNF expression	[150]
	Male C57BL/6N mice induced by MPTP and 6-OHDA	GV20, GV14	EA, 1 mA, 2 Hz, 20 min, QD for 10 d	Induce the expression of CREB/AKT/Pitx3 in dopaminergic neurons	Attenuate dopaminergic neuron loss in the SN, up-regulate BDNF and GDNF expression in both the SN and striatum	[151]
	Male C57BL/6N mice induced by MPTP	GB34, LR3	EA, 1 mA, 0/50 Hz, 20 min, QD for 8 d	Activation of the AKT pathway	Improved striatal dopamine levels	[152]
	SD rats induced by 6-OHDA	GV20, GV14	EA, 3 mA, 100 Hz, 30 min, 6 d a week for 4 wk	Increase BDNF and Trk-B in the midbrain and hippocampus	Alleviate depressive-like symptoms	[153]
	Male C57BL/6N mice induced by MPTP	GB34	MA, 2 spins per second for 15 s, QD for 7 d	Activation of the AKT pathway	Dopaminergic neuron protection and motor function improvement	[154]
	Male SD rats induced by intradermal-injection of rotenone	GV16, LR3	EA, 2 mA, 2 Hz, 20 min, QD for 14 d	Down-regulate p-ERK 1/2, TNF-α and IL-1β proteins	Improve PD movement	[155]
Depression	Male SD rats, CUMS	GV20, GV29, LI4, LR3	EA, 1–1.2 mA, 2/100 Hz, 20 min, QD for 14 d	Up-regulate BDNF, mTORC1 expression in prefrontal cortex	Up-regulate PSD95, SynapsinⅠ, and GluR1 expression in prefrontal cortex, and elevate immature spine dendritic densities	[156]
	Male SD rats, CUMS	GV20, GV29	EA, 2 mA, 2 Hz, 20 min, QD for 28 d	Increase the levels of tPA, BDNF, Trk-B, and BDNF micro RNA in hippocampus	Improve depressive-like behaviors in CUMS	[157]
	Male SD rats, CUMS	GV20, GV29	MA, 10 min, QD for 21 d	Up-regulate BDNF/p-ERK 1/2 expression	Improve depressive-like behaviors in CUMS	[158]
	Male SD rats, CUMS	GV20, GV29	EA, 0.6 mA, 2 Hz, 30 min, QD for 14 d	Up-regulate BDNF/ Trk-B/CREB expression	Improve depressive-like behaviors in CUMS	[159]
	Male SD rats, CUMS	GV20, GV29	EA, 2 Hz, 30 min, QD for 3 wk	Increase BDNF and Trk-B in hippocampus	EA combined with a low dose of citalopram could produce greater therapeutic effects	[160]
	Male Wistar rats subjected to repeated alcohol administration	HT7	MA, stimulate the HT7 point for 30 s and hold in place for up to 1 min, QD for 2 wk	Increase the amygdala expression of BDNF and Trk-B	Alleviate increased stress hormone levels and mitigate anxiety and negative emotions	[161]
	PSD SD rats modeled with MCAO combined with CUMS	LI4, LR3	EA, 2/20 Hz, 30 min, QD for 21 d	Elevate the numbers of BDNF- and Trk-B-positive cells in hippocampus	Improve depressive-like behaviors	[162]
	PSD SD rats modeled with MCAO combined with CUMS	GV14, GV26, GV20, GV24	MA, 40 min, QD, 6 times a week for 4 wk	Increase the expression of PI3K/AKT/mTOR	Improve depression-like behavior in PSD rats	[163]
	Male SD rats, CUMS	GV20, GV29	MA, 10 min, QD for 21 d	Up-regulated p-ERK 1/2 and BDNF expression	Improve depression-like behavior	[158]
	Male SD rats, CUMS	GV20, GV29	MA, at a frequency of twice per second for 1 min, retained for 10 min, QD for 3 wk	Up-regulate BDNF/ERK/CREB expression	Ameliorate the depression-like behaviors and dysfunction of the ERK signaling pathway	[164]
	Depressed Bagg albino (BALB/c) mice induced by chronic neuropathic pain	ST36, GB34	EA, 1.5 mA, 2/100 Hz, 30 min, QD for 7 d	Up-regulate BDNF/CREB expression	Up-regulation of BDNF and 5-HT expression	[165]

6-OHDA = 6-hydroxydopamine, Aβ = amyloid-beta, AD = Alzheimer disease, AKT = protein kinase B, AMPK = adenosine monophosphate-activated protein kinase, APP/PS1 = amyloid precursor protein/presenilin-1, Bax = Bcl-2 associated X, Bcl-2 = B-cell lymphoma-2, BDNF = brain-derived neurotrophic factor, CCAO = occlusion of common carotid artery, CaM = calmodulin, CaMKIV = Ca2+/calmodulin-dependent protein kinase type IV, CHT1 = choline transporter 1, CIRI = cerebral ischemia; Reperfusion injury, CREB = CAMP-related element-binding protein, CUMS = chronic unpredictable mild stress, CV24 = Chengjiang acupoint, ERK = extracellular signal-regulated kinase, GAP-43 = growth associated protein-43, GDNF = glial-derived neurotrophic factor, Klf4 = Krüppel-like factor 4, LCCA = left common carotid artery, LI4 = Hegu acupoint, LTP = long-term potentiation, MCBVAO = MCAO plus bilateral vertebral artery occlusion, MPTP = 1-methyl-4-phenyl-1,2,3,6-tetrahydropyridine, MSCs = mesenchymal stem cells, NDs = neurodegenerative diseases, NF-𝜅B = nuclear factor kappa-light-chain-enhancer of activated B cells, NO = nitric oxide, nNOS = neuronal NO synthase, iNOS = inducible NO synthase, NT = neurotrophin, p38 MAPKs = p38 mitogen-activated protein kinases, PI3K = phosphatidylinositol-4,5-bisphosphate 3-kinase, p75NTR = pan-neurotrophin receptor p75, PD = Parkinson disease, PFC = prefrontal cortex, PKA = protein kinase A, PSD = poststroke depression, SN = substantia nigra, SVZ = subventricular zone, TLR4 = toll-like receptor 4, TREM2 = triggering receptor expressed on myeloid cells 2, TrK-B = tropomyosin-related kinase B, Trk-B-MSCs = Trk-B gene-transfected mesenchymal stem cells, VAChT = vesicular acetylcholine transporter.

### 4.1. Stroke

#### 4.1.1. Acupuncture increases BDNF to improve neuronal function in stroke

Accumulating research demonstrates that acupuncture intervention up-regulates BDNF expression to improve neurogenesis and promote functional recovery in ischemic stroke animal models. Acupuncture promotes neurogenesis, neuronal stem cells proliferation and differentiation and axonal regrowth after hypoxic-ischemic insults through increasing BDNF and other neuroplasticity-related proteins such as vascular endothelial growth factor, glial-derived neurotrophic factor (GDNF) and GAP-43 within the central nervous system(CNS).^[166–169]^ EA applied to Quchi (LI11) and Zusanli (ST36) acupoints on the contralateral paralyzed limb markedlyameliorates neurological impairments and diminishes cerebral infarct volume, while concurrently elevating serum concentrations of BDNF and GDNF.^[170]^ EA intervention at these acupoints confers neuroprotective effects via proliferation of glial fibrillary acidic protein/vimentin/nestin-positive reactive astrocytes and the potential release of astrocyte-derived BDNF in CIRI models.^[14]^ The combination of scalp acupuncture and cognitive rehabilitation effectively boosts BDNF and nerve growth factor levels in stroke patients’plasma, concurrently ameliorating cognitive performance and motor capabilities throughout the recovery phase.^[171]^ Additionally, EA targeting trigeminal nerve-innervated acupoints alleviates poststroke cognitive dysfunction by counteracting the CIRI-induced downregulation of BDNF, Trk-B, and N-methyl-D-aspartate receptor (NMDAR) expression in MCAO rats.^[172]^ Furthermore, EA at Baihui (GV20) acupoint promotes motor function recovery and up-regulates BDNF/Trk-B signaling pathways in experimental cerebral ischemia models.^[119,123,173]^ The 2Hz EA treatment increases BDNF and improves dysphagia after stroke in mice.^[174]^ Therapeutic acupuncture targeting acupoints Dazhui (GV14), Fengfu (GV16), Shenting (GV24), Shendao (GV11), Baihui (GV20), and Shuigou (GV26), combined with standardized swallowing rehabilitation demonstrates significant efficacy in poststroke dysphagia management. This integrated intervention enhances swallowing function, improves cerebral perfusion, and up-regulates serum levels of BDNF and nerve growth factor in patients with ischemic stroke.^[175]^ EA combined with grafted Trk-B gene-transfected mesenchymal stem cells (Trk-B-MSCs) up-regulates the expression of BDNF and neurotrophin(NT)-4/5, induces Trk-B-MSC differentiation, and improves locomotor function in ischemic stroke mice.^[120]^ Combined MSCs and EA at GV20 and GV14 treatment leads to an increase in BDNF, NT-4 and phosphorylated CREB protein in the neuroblasts of the striatum neurotrophic factors to improve motor function in MCAO mice.^[15]^ A recent study demonstrates that integrative therapy involving scalp acupuncture and physical exercise mitigates ischemic cerebral damage in rats not only via increasing BDNF and SOD production, but also via downregulating pro-inflammatory mediators.^[122]^

Collectively, these research findings indicate that acupuncture-based interventions ameliorate neurological deficits and cerebral infarcts. The underlying mechanism for EA’s efficacy in ischemic stroke management may be attributed to the elevation of BDNF protein concentrations in both neural tissues and systemic circulation.

#### 4.1.2. *Acupuncture regulates miRNAs to improve neuronal function in stroke*

By performing RNA sequencing and a protein–protein interaction network in MCAO mice, Wu et al identified 53 up-regulated and 121 downregulated genes in EA-treated groups compared to controls, associated with the forkhead box O (FOXO) signaling pathway, noncanonical nuclear factor-kappaB (NF-κB) signaling pathway, T-cell receptor signaling pathway, and other vital pathways. Key miRNA-mRNA regulatory networks, such as mmu-miR-434-3p-Prc1, mmu-miR-453-Prc1, miR-425-5p-Cdk1, and mmu-miR-1186b-Prc1, were delineated as central modules modulated by EA and implicated in ischemic stroke pathogenesis.^[103]^ Notably, mmu-miR-434-3p is postulated to enhance neuronal regeneration under stress conditions, particularly following cerebral injury.^[176–178]^ The miR-453 demonstrates strong correlations with favorable neural repair outcomes during postischemic recovery.^[179,180]^ Moreover, mmu-miR-425-5p attenuates apoptotic processes and suppresses necroptosis, oxidative stress, and inflammatory responses via inhibition of the receptor-interacting protein kinase 1 signaling axis.^[181,182]^ Furthermore, EA facilitates the differentiation of endogenous neural stem cells (NSCs) through exosome-mediated transfer of miR-146b, thereby ameliorating neurological deficits postischemic stroke.^[104]^ In a CIRI rat model, EA at the Waiguan (TE5) and ST36 acupoints promotes miR-223 expression to reduce Phosphatase and tensin homolog(PTEN) expression so that increases the number of NSCs.^[105]^ Following electroacupuncture intervention, up-regulation of miR-132 inhibits the expression of sex-determining region Y-box 2 (SOX2) in primary neurons subjected to OGD, thereby facilitating neurite extension and outgrowth.^[106]^ EA treatment mitigates ischemic brain injury by downregulating neuronal calcium sensor 1 via miR-191a-5p targeting in cerebral ischemia modelszz.^[107]^ EA at Shuigou (GV26) and Neiguan (PC6) modulates the expression profiles of rno-miR-206-3p, rno-miR-3473, rno-miR-6216 and rno-miR-494-3p which are related with the vascular endothelial growth factor signaling pathway linking cell proliferation and improve cerebral blood supply and functional recovery in a rat model of stroke.^[108]^ EA at GV20 and GV24 acupoints miR-134 expression, which negatively regulates LIM domain kinase 1 (LIMK1) activity, thereby enhancing synaptic-dendritic plasticity and improving learning and memory capabilities during the recovery phase of ischemic stroke.^[183]^ EA at GV20 elevates miR-181b expression in ischemic penumbral regions and promotes neurobehavioral recovery through miR-181b-mediated targeting of paired immunoglobulin-like receptor B (PirB) mRNA, subsequently modulating the expression of PirB, RhoA, and GAP-43 in MCAO models.^[109]^

Acupuncture not only regulates microRNA to improve neuronal function but also regulates microRNA to affect neuroinflammation. EA treatment can alleviate neuroinflammation by inhibiting the miR-223/ nucleotide-binding domain-like receptor family (NLRP3) pathway, thereby exerting neuroprotective effects in MCAO in rats.^[110]^ Stimulation of LI11 and ST36 acupoints with electroacupuncture up-regulates miR-9 expression in the peri-infarct cortical regions, leading to inhibition of NF-κB signaling and subsequent attenuation of inflammatory damage in MCAO rats.^[111]^ GV20-penetrating-Qubin (GB7) acupuncture decreases miR-34a-5p levels which inhibits microglia M2 polarization while promoting M1 polarization via targeted regulation of Krüppel-like factor 4 (Klf4).^[112]^

#### 4.1.3. Acupuncture regulates downstream neuropathways of BDNF to improve neuronal function in stroke

Acupuncture on GV20 elevates BDNF level, increases expression of BDNF/Trk-B and induces neurogenesis in experimental models of cerebral ischemia.^[184]^ In parallel, electroacupuncture application at the LI11 and ST36 acupoints demonstrates neuroprotective efficacy neuroprotective role in CIRI rats by modulating the PI3K/AKT^[184]^ pathway.^[170]^ Acupuncture promotes neurovascular unit repairment in cerebral infarction models through modulating the PI3K/AKT signaling pathway.^[125]^ Xie et al demonstrates that EA intervention ameliorates neurological deficit scores, up-regulates p-AKT protein levels, and enhances the percentage of bone marrow-derived CD34 + endothelial progenitor cells in CIRI rats.^[126]^ Accumulating evidence indicates that EA application at specific acupoints, including GV20, GV24, GV26, Yongquan(KI1), LI11, and ST36, stimulates the PI3K/AKT signaling cascade, conferring antiapoptotic and neuroprotective efficacy.^[127,129–131,185,186]^ The EA modulation of the PI3K pathway initiates downstream activation of the mTOR complex 1–UNC-51-like kinase 1 axis, reduces the expression levels of caspase-3, caspase-8, and caspase-9, and suppresses autophagic flux.^[132]^ Stimulation of GV24 and GV20 acupoints with EA activates the PI3K/AKT/mTOR signaling pathway, leading to improved cognitive function in rats subjected to cerebral ischemic events.^[133]^ EA also attenuates nitric oxide (NO) production by downregulating neuronal NO synthase (nNOS) and inducible NO synthase (iNOS) expression through PI3K pathway activation.^[130]^ Genetic silencing of triggering receptor expressed on TREM2 impairs the capacity of electroacupuncture to modulate both PI3K/AKT and NF-κB signaling pathways, thereby diminishing its anti-inflammatory effects in CIRI.^[134]^ EA profoundly activates the PI3K/AKT signaling pathway, resulting in increased p-Bad, elevated Bcl-2 expression, and inhibition of Bax and cleaved caspase-3, collectively suppressing neuronal apoptosis within the ischemic penumbra of CIRI rats.^[135]^

EA can also trigger the MAPK family, with ERK pathway activation being closely associated with elevated BDNF expression.^[136]^ EA can reduce ischemic brain infarct volume and enhance neurological recovery via activation of the ERK1/2 signaling cascade.^[124,136–138]^ EA demonstrates efficacy in promoting neuronal survival and stimulating neural cell proliferation, as evidenced by multiple experimental studies. The CREB pathway interacts with multiple factors, including BDNF, p38 MAPK, and Ca2+/calmodulin-dependent protein kinase (CaMK), forming a complex regulatory network.^[139–141]^ Acupuncture stimulation at specific acupoints, including GV16, GV20, GV24, ST36, and Shenmen(HT7), activates hippocampal CREB signaling and ameliorates cognitive deficits in neurological disorder models.^[139,140,142,143]^ EA exertes neuroprotections against neuronal apoptosis and the mechanism may be involved in up-regulation of the growth factor midkine (MK) and mediation of ERK/JNK/p38 signal pathway.^[144]^ Another research shows that acupuncture and hypothermia therapy can reduce p-MEK2 and p-ERK1/2 level to down-regulate Bax and up-regulate Bcl-2, thus improve cerebral function in CIRI rats.^[145]^

### 4.2. AD

#### 4.2.1. Acupuncture increases BDNF to improve neuronal function in AD:

EA may represents a potentially viable therapeutic intervention for AD, demonstrating neuroprotective efficacy through modulation of BDNF expression. Therapeutic Sanjiao acupuncture targeting specific acupoints – including Danzhong (CV17), Zhongwan (CV12), Qihai (CV6), bilateral Xuehai (SP10), and bilateral Zusanli (ST36) – effectively regulates concentrations of key cytokines such as fibroblast growth factor-basic (bFGF), epidermal growth factor, and BDNF within the hippocampal microenvironment. This cytokine modulation enhances NSCs survival, proliferation, and differentiation capacities, thereby facilitating repair of compromised neural cells and ultimately improving cognitive function in AD mice.^[187]^ EA at GV 20, GV 16 and Shenshu(BL23) can activate the proliferation of NSCs in the hippocampus in AD mice contributes to upregulating the expression of BDNF.^[188]^ Li and colleagues demonstrate that repeated electroacupuncture stimulation ameliorates cognitive performance, up-regulates BDNF expression, and enhances hippocampal neurogenesis in AD.^[189]^ Acupuncture intervention on GV20 acupoint significantly mitigates Aβ deposits, elevates BDNF protein levels, and confers substantial neuroprotective efficacy on CNS cells.^[190,146]^

#### 4.2.2. Acupuncture regulates miRNAs to improve neuronal function in AD

Keifer and colleagues conducted a study focused on elucidating the interconnected miRNA-BDNF signaling circuitry within the AD brain. Their findings reveal that BDNF deficiency in AD pathogenesis arises from 2 distinct molecular mechanisms: impaired proteolytic processing of pro-BDNF into biologically active mature BDNF, and miRNA-mediated posttranscriptional repression of BDNF gene expression.^[146]^ Consequently, the regulatory influence of miRNAs on BDNF signaling must be integrated into the development of BDNF-targeted acupuncture therapies for AD. Complementarily, DNA microarray profiling indicates that Sanjiao acupuncture intervention reverses aging-associated transcriptional alterations in the hippocampal region of senescence-accelerated mouse prone 10 (SAMP10) models and attenuates oxidative stress-mediated cellular damage.^[113]^ Furthermore, N-myc downregulated gene 2 (NDRG2) encodes a cytosolic protein implicated in neuritogenesis. EA application at GV20 acupoint suppresses the astrocyte NDRG2 expression and glial fibrillary acidic protein level, thus improving memory impairment of amyloid precursor protein/presenilin-1 (APP/PS1) double transgenic mice.^[114]^

#### 4.2.3. Acupuncture regulates downstream neuropathways of BDNF to improved neuronal function in AD

Aβ deposition disrupts key BDNF-associated signaling cascades, including the Ras/ERK, PI3K/AKT, and protein kinase A/cyclic adenosine monophosphate (PKA/cAMP) pathways, which collectively regulate BDNF expression and contribute to AD pathogenesis.^[191,192]^ Lin et al reports that elevation BDNF–Trk-B pathway exerts potent antiapoptotic effects in APP/PS1 mice.^[146]^ Lee et al reports that acupuncture intervention potentiates the cholinergic system–CREB–BDNF signaling axis, a critical pathway governing learning processes and synaptic plasticity in limbic circuitry, thereby conferring significant neuroprotective benefits.^[190]^ Telomerase, a crucial enzyme implicated in aging and apoptotic processes, is modulated by acupuncture intervention. Research by Lin and colleagues shows that acupuncture stimulation at Dubi (ST35) acupoint in telomerase-deficient murine models activates the BDNF-Trk-B pathway, concurrently upregulating expression levels of BDNF, Trk-B, AKT, and ERK1/2 expression, ultimately enhancing telomerase activity.^[148]^ Acupuncture intervention on GV20 acupoint up-regulates glucose transporter (GLUT1 and GLUT3), p-AMPK, p-AKT, and mTOR levels in hippocampal and cortical regions. By modulating cerebral energy metabolism, this intervention reduces Aβ deposition, suppresses autophagic processes, and ameliorates cognitive deficits.^[149]^

### 4.3. PD

#### 4.3.1. Acupuncture increases BDNF to improve neuronal function in PD

EA treatment up-regulates BDNF and GDNF mRNA expression in the SN of Parkinson rat models.^[193,194]^ In experimental PD rats, EA therapy confers robust neuroprotection for dopaminergic neurons through enhanced expression of BDNF, GDNF, and associated signaling molecules, consequently ameliorating motor dysfunctio.^[151]^ EA inhibits apomorphine induced rotational behavior and locomotor activity, while exhibiting neuroprotective efficacy through activation of the BDNF/AKT survival signaling axis and in the SN region.^[152]^ EA up-regulates BDNF expression and restores Trk-B neurotrophic signaling to ameliorate motor dysfunction and restores the dopaminergic neuronal function in MPTP-lesioned mice.^[150]^ The combined therapy of EA and medication demonstrates synergistic efficacy in modulating serum BDNF concentrations and alleviating depressive symptoms in PD patients.^[195]^ Furthermore, comparative investigations by Sun et al and Liang et al reveal distinct frequency-dependent outcomes of chronic electroacupuncture stimulation in PD rat models. Research demonstrates that a 4-week regimen of 100 Hz electroacupuncture therapy effectively ameliorates aberrant expression of BDNF within the lesioned ventral midbrain and hippocampal regions induced by 6-OHDA. Previous investigations have established the efficacy of EA in ameliorating motor dysfunction in PD models. Nonetheless, the therapeutic potential and mechanistic basis of EA in addressing PD-related depressive manifestations remain inadequately elucidated. In this study, a rodent model of PD was established through unilateral 6-OHDA lesions targeted at the medial forebrain bundle, followed by a 4-week EA intervention. We observed that 100 Hz EA administration significantly ameliorated multiple motor phenotypes in these animals. Immunohistochemical assessment of tyrosine hydroxylase (TH) revealed that EA exerted minimal effects on TH-positive neuronal profiles within the ipsilateral ventral tegmental area. Furthermore, compared with 6-OHDA lesioned controls, prolonged EA stimulation markedly enhanced sucrose preference test consumption and reduced forced swim test immobility periods, indicating mitigation of depression-like behaviors. Notably, EA intervention did not significantly produce significant alterations in concentrations of dopamine, norepinephrine, or serotonin within the striatal or hippocampal regions. However, EA effectively normalized the 6-OHDA-induced aberrant expression patterns of BDNF and its high-affinity receptor Trk-B in both the midbrain and hippocampal formations. These findings demonstrate that 100 Hz EA ameliorates depressive-like manifestations in PD models, partially through selective modulation of mesostriatal and mesocorticolimbic dopaminergic pathways. BDNF appears to be critically involved in this EA-mediated effect.^[153]^ Furthermore, BDNF mRNA levels were significantly elevated in the SN and ventral tegmental area of the lesioned hemisphere in the 100 Hz EA group, with no changes observed in the 0 Hz or 2 Hz groups. Long-term high-frequency EA facilitates the regeneration of impaired dopaminergic neurons by activating endogenous BDNF signaling pathways.^[196]^

#### 4.3.2. Acupuncture regulates miRNAs to improve neuronal function in PD

Research findings have indicated that miR-124 exhibits significant pathogenic involvement in PD progression.^[197]^ Genetic knockdown of CDK5 demonstrates therapeutic potential by upregulating BDNF expression and ameliorating synaptic impairments in PD models. Overexpression of miR-124 can silence CDK5 activity through inhibition of calpain1/p25/CDK5 signaling cascade.^[46]^ Liu finds that acupuncture applied to acupoints Yanglingquan(GB34) and Taichong(LR3) can up-regulate miR-124 expression within the striatal region of PD transgenic murine models.^[115]^

#### 4.3.3. Acupuncture regulates downstream neuropathways of BDNF to improve neuronal function in PD

Acupuncture-mediated stimulation potentiates PI3K/AKT signaling cascade activation, thereby improving dopaminergic turnover dynamics and synaptic efficacy within the SN and striatal synaptic regions, while concurrently modulating the cell cycle progression of TH-positive neurons, ultimately resulting in significant improvement of motor function.^[152,154,198]^ EA stimulation at 2 Hz applied to GV16 and LR3 acupoints modulates key signal transduction pathways by inactivating both ERK 1/2 signaling pathway and p38/MAPK signaling pathway. This intervention promotes the proliferation of TH-positive neurons while simultaneously reducing the expression of pro-inflammatory mediators including cyclooxygenase (COX)-2, tumor necrosis factor-𝛼, and interleukin(IL)-1𝛽 levels, thereby effectively alleviating PD symptomatology.^[199,155]^

### 4.4. Depression

#### 4.4.1. Acupuncture increases BDNF to improve neuronal function in depression

EA is widely recognized as a secure and noninvasive therapeutic modality for mitigating depressive symptoms, while concurrently ameliorating depression-associated impairments in long-term potentiation.^[200]^ Reactive and endogenous depression represent etiologically heterogeneous subtypes that exhibit divergent responses to antidepressant interventions. Treatment strategies should be refined through precise identification of the depressive subtype to enhance therapeutic efficacy. Yang et al identify a significant inverse correlation between BDNF levels and Hamilton Depression Rating Scale scores in individuals with reactive depression, suggesting BDNF as a promising biomarker for detecting and assessing the severity of this subtype.^[201]^ EA attenuates depressive manifestations by upregulating key components of the BDNF signaling cascade, augmenting dendritic spine density, and fostering synaptic plasticity within the prefrontal cortex.^[156–160,202–204]^ Concurrent acupuncture stimulation at GV20 and Yintáng(GV29) acupoints over a 2-week period reverses social defeat stress-induced reductions in BDNF, NT-3, and NT-4/5 expression, an effect not elicited by conventional antidepressant medications.^[205]^ The antidepressant efficacy of acupuncture may involve epigenetic mechanisms, including modulation of DNA methylation and histone modifications at the BDNF gene locus.^[206]^ Besides BDNF, acupuncture alleviates depression-like behaviors by modulating gut microbial communities and adjusting neurotransmitter dynamics, encompassing dopamine, serotonin (5-HT), corticosterone, and sex hormone concentrations in both serum and hippocampal tissues.^[161,207–210]^ Other therapeutic mechanisms of acupuncture include inhibition of hypothalamic-pituitary-adrenal (HPA) axis hyperactivity and inflammation, modulation of the expression of particular genes, etc.^[211]^

#### 4.4.2. Acupuncture regulates miRNAs to improve neuronal function in depression

Duan used microarray to investigate alterations in miRNA expression patterns in response to depression and EA intervention in CUMS rats. Their analysis revealed that miR-383-5p and miR-764-5p expressions were significantly elevated following CUMS exposure but markedly downregulated after EA treatment. These findings suggest that EA may confer antidepressant effects through enhancing neurotrophic support, suppressing aberrant neuronal apoptosis, and modulating associated signaling pathways.^[116]^ miR-16 demonstrates a close association with depression pathogenesis, with experimental evidence indicating that it directly targets 3′-UTR of BDNF transcripts.^[67]^ EA application at acupoints GV20 and GV29 effectively attenuates elevated miR-16 expression and ameliorates depression-like behaviors in in CUMS rat model.^[117]^ Mongolian medicinal 3-acupoint balance needling technique alleviates depressive-like behaviors by regulating the p11/tPA/BDNF signaling axis and modulating miR-16 expression in both the hippocampal formation and the median raphe nucleus of depression model rats.^[118]^

#### 4.4.3. Acupuncture regulates downstream neuropathways of BDNF to improve neuronal function in depression

Integrated bioinformatics analyses utilizing GO and KEGG pathway enrichment indicates that acupuncture treats depression through regulating multiple signaling cascades, including the Toll-like receptor signaling pathway, NLRP3 inflammasome, MAPK/ERK, PI3K/AKT, neurotrophin, tumor necrosis factor, and NF-𝜅B pathways. The aforementioned pathways collectively regulate critical cellular processes such as neuronal survival, differentiation, antiapoptotic mechanisms, and synaptic plasticity mechanisms, thereby attenuating depressive symptomatology and mitigating learning and memory impairments associated with depression.^[116,212,213]^ EA at Siguan acupoints demonstrates superior efficacy compared to fluoxetine in alleviating depressive behaviors in poststroke depression (PSD) rats, potentially through up-regulation of BDNF and its receptor Trk-B.^[162]^ Acupuncture stimulation at acupoints at GV14, GV26, GV20 and GV24 can demonstrate efficacy in ameliorating depression-like behavioral phenotypes in PSD rats by activation PI3K/AKT/mTOR signaling axis and suppression hippocampal neuron autophagy.^[163]^ Striatal-enriched tyrosine phosphatase (STEP) exerts its pathological effects through dephosphorylation and inactivation of key signaling molecules including ERK1/2, p38 MAPKs, and Src family tyrosine kinases. This enzymatic activity leads to downregulation of BDNF expression, triggering progressive neuronal degeneration in hippocampal and cortical regions, thereby elevating susceptibility to depressive disorders.^[214]^ Acupuncture treatment demonstrates significant antidepressant efficacy in CUMS-induced depression models. This therapeutic effect is mediated through up-regulation of p-ERK1/2 and enhanced BDNF expression in the prefrontal cortex, suggesting a crucial role for ERK-BDNF signaling in acupuncture’s mechanism of action.^[155,164,215–217]^ Substantial evidence indicates that acupuncture stimulation activates the MAPK/ERK signaling cascade, subsequently triggering CREB pathway activation, which collectively contributes to the amelioration of depressive states.^[218,219]^ The most frequently selected acupoints for depressive disorders are GV20 and GV29, with supplementary use of GB34 and PC6. EA produces concurrent analgesic and antidepressant effects by modulating the CREB-serotonin-BDNF signaling axis in the anterior cingulate cortex and spinal cord regions in murine models of chronic neuropathic pain comorbidity with depression-like behaviors.^[165]^

## 5. Mechanistic insights into acupuncture regulation of BDNF and miRNAs

While increasing evidence underscores acupuncture’s role in modulating BDNF and microRNA (miRNA) expression, the precise molecular mechanisms driving these regulatory effects remain to be fully elucidated. Multiple interconnected pathways have been proposed to explain acupuncture’s therapeutic actions.

Primarily, acupuncture stimulation potentially activates cutaneous and muscular afferent nerve fibers, such as Aδ and C fibers, initiating signal propagation through spinal pathways to key brain regions including the hippocampus, hypothalamus, and cerebral cortex. These areas are pivotal for neurotrophin production and secretion, particularly BDNF. Research indicates that acupuncture enhances neuronal excitability and calcium signaling in these regions, subsequently triggering the cAMP response element-binding protein (CREB) pathway, a crucial transcriptional activator of BDNF gene expression.^[121]^

Additionally, acupuncture demonstrates modulatory effects on the HPA axis, attenuating stress-associated hormone levels including corticosterone, which normally suppresses BDNF synthesis. Through this stress-reduction mechanism, acupuncture indirectly promotes neurotrophin availability and supports neurogenesis.^[128]^

Furthermore, epigenetic mechanisms may mediate acupuncture’s miRNA regulatory effects, involving alterations in histone acetylation states and DNA methylation patterns within miRNA gene promoter regions. Such modifications can influence miRNA transcriptional activity related to neuroplasticity, inflammatory responses, and apoptotic processes.^[147]^ Notably, specific miRNAs like miR-132 and miR-146 exhibit co-regulation with neurotrophic signaling pathways, responding to neuronal activity while modulating synaptic protein and neurotrophic factor expression.^[104,220]^

Finally, emerging research also suggests acupuncture influences exosomal miRNA profiles, enabling intercellular communication among neurons, astrocytes, and microglia. These exosomal miRNAs potentially target BDNF pathway components, facilitating neural repair and synaptic reorganization.^[221,222]^

Collectively, acupuncture appears to regulate BDNF and associated miRNAs through integrated mechanisms encompassing neural activation, neuroendocrine adjustment, epigenetic modification, and intercellular signaling. Comprehensive investigations combining electrophysiological, transcriptomic, and epigenomic methodologies are essential to delineate the molecular foundations of acupuncture-induced neuroplastic changes.

## 6. Conclusion

Rooted in traditional Oriental medical theory, acupuncture practice operates through a meridian network of 14 primary channels connecting approximately 360 identified acupoints. Although this theoretical framework is robustly established, the specific neurobiological mechanisms underlying acupuncture’s therapeutic efficacy remain incompletely elucidated. Emerging evidence indicates that needling application at precise cutaneous acupoints alters peripheral neural signaling, inducing neurophysiological modifications within the CNS that ultimately facilitate clinical therapeutic outcomes. BDNF modulates synaptic plasticity mechanisms, thereby supporting motor learning, rehabilitation, and cognitive functions such as learning and memory. In contrast, diminished BDNF expression and disrupted signaling pathways are implicated in the pathogenesis of neurodegenerative disorders. Acupuncture can increase BDNF and regulate miRNAs which can control BDNF expression to enhance neural plasticity, survival, NSC proliferation and neuronal differentiation via BDNF downstream signaling including PI3K/AKT, MAPK/ERK and PLCγ. Acupuncture represents a cost-effective therapeutic intervention with a favorable safety profile, facilitating its integration with complementary treatment modalities for neurodegenerative disorder.

## Acknowledgments

The authors would like to thank all the participants in our study.

## Author contributions

**Conceptualization:** Jingxin Li.

**Data curation:** Lijuan Liu.

**Formal analysis:** Shuang Sun, Chunyan Liu.

**Writing – original draft:** Jingxin Li.

**Writing – review & editing:** Ping Yin.
